# Assessing the Genetic Diversity of Austrian Corynebacterium diphtheriae Clinical Isolates, 2011 to 2019

**DOI:** 10.1128/JCM.02529-20

**Published:** 2021-02-18

**Authors:** Justine Schaeffer, Steliana Huhulescu, Anna Stoeger, Franz Allerberger, Werner Ruppitsch

**Affiliations:** a Institute for Medical Microbiology and Hygiene, Austrian Agency for Health and Food Safety, Vienna, Austria; b EUPHEM Fellowship, European Centre for Disease Prevention and Control (ECDC), Stockholm, Sweden; National Institute of Allergy and Infectious Diseases

**Keywords:** *Corynebacterium diphtheriae*, whole-genome sequencing, core genome MLST, antibiotic resistance, diphtheria toxin

## Abstract

Diphtheria is a vaccine-preventable disease with a high potential for reemergence. One of its causative agents is Corynebacterium diphtheriae, with some strains producing diphtheria toxin. From 2011 to 2019, 57 clinical C. diphtheriae strains were isolated in Austria, either from the respiratory tract or from skin infections. The aim of this study was to investigate the genetic diversity of these C. diphtheriae isolates using whole-genome sequencing. Isolates were characterized by genome-wide comparisons using single nucleotide polymorphism analysis or core genome multilocus sequence typing and by searching sequence data for antimicrobial resistance genes and genes involved in diphtheria toxin production. The genetic diversity among the isolates was high, with no clear distribution over time or place. Corynebacterium belfantii isolates were separated from other strains and were strongly associated with respiratory infections (odds ratio [OR] = 57). Two clusters, limited in time and space, were identified. Almost 40% of strains carried resistance genes against tetracycline or sulfonamides, mostly from skin infections. Microbiological tests showed that 55% of isolates were resistant to penicillin but did not carry genes conferring β-lactam resistance. A diphtheria toxin gene with no nonsynonymous mutation was found in three isolates only. This study showed that sequencing can provide valuable information complementing routine microbiological and epidemiological investigations. It allowed us to identify unknown clusters, evaluate antimicrobial resistance more broadly, and support toxigenicity results obtained by PCR. For these reasons, C. diphtheriae surveillance could strongly benefit from the routine implementation of whole-genome sequencing.

## Abstract (precis)

Diphtheria is a vaccine-preventable disease with a high potential for reemergence. One of its causative agents is Corynebacterium diphtheriae, with some strains producing diphtheria toxin.

## INTRODUCTION

Corynebacterium diphtheriae and, more rarely, C. ulcerans and C. pseudotuberculosis are the causative agents of diphtheria, a severe toxin-mediated disease. Nasopharyngeal or cutaneous infection by toxigenic *Corynebacterium* is associated with diphtheric lesions (necrotic tissues). Systemic dissemination can lead to paralysis, myocarditis, and nephritis (1). The death rate is high, up to 50% without treatment and still up to 10% with antitoxin and antibiotic therapy (2). C. diphtheriae is efficiently transmitted from person to person through droplets, secretions, or direct contact. This combination of high severity and easy transmission makes C. diphtheriae a pathogen of major public health importance.

In low-income countries, diphtheria is still a significant burden (3). Crises leading to the breakdown of the health care system favor diphtheria reemergence, such as the collapse of the Soviet Union, the Venezuelan economic crisis, or the Rohingya refugees in Bangladesh (4–6). In high-income countries, vaccination plans have reduced diphtheria cases to sporadic events, with fewer than 70 cases per year in European Union (EU) and EU-associated countries (7). However, as protection among the vaccinated population tends to drop over time, the potential for outbreaks is high (3). The European refugee crisis has also been associated with an increased diphtheria incidence, especially in migrant camps (8).

C. diphtheriae can become toxigenic by acquiring the diphtheria toxin (DT) gene (*tox*) through corynebacteriophage lysogenization (9–11). Carriage of *tox*-negative *Corynebacterium* has been shown, but its prevalence is still unknown (12). There is the potential for *in situ* phage conversion of *tox*-negative strains, even though it was never proven. Even without converting to toxigenicity, some nontoxigenic C. diphtheriae strains can also cause severe disease in both vaccinated and unvaccinated patients, with a mortality rate of up to 40% (12, 13). Therefore, even in countries with high vaccination coverage, close monitoring of C. diphtheriae is still necessary.

Every C. diphtheriae strain isolated in Austria since 2011 has been kept in the diphtheria national reference center (NRC) collection. So far, the only information available about Austrian C. diphtheriae isolates was *tox* status and biovar (based on metabolic properties). The aim of this study was to characterize these isolates using whole-genome sequencing (WGS) in order to assess C. diphtheriae diversity in Austria over the past decade. In addition, even though *Corynebacterium* infections have to be reported, few epidemiological data were available on the cases. Sequence data might be a way to complement these data and inform C. diphtheriae surveillance.

## MATERIALS AND METHODS

### Samples.

Suspected *Corynebacterium* samples (wound swabs or pus, respiratory tract swabs, respiratory tract liquid, or blood) were sent to the Austrian NRC for diphtheria for diagnosis. Data used in this study fall within the mandate given to the NRC by the Austrian Ministry of Health and did not require additional ethical approval. Bacteria were cultured on Hoyle’s agar (Becton, Dickinson, Heidelberg, Germany). The biochemical test api Coryne (bioMérieux, Marcy-l’Etoile, France) was used to assign isolates to C. diphtheriae bv. *gravis* or C. diphtheriae bv. *mitis*/bv. *belfanti*. As has been proposed by Dazas et al., isolates previously reported as C. diphtheriae bv. *belfanti* are referred to as C. belfantii in this article (14). Positive isolates were kept at −80°C in the NRC collection. *tox* status was determined using reverse transcription-quantitative PCR (RT-qPCR) targeting subunits A (SubA) and B (SubB) of DT (15). Antimicrobial resistance (AMR) against penicillin G, clindamycin, rifampicin, vancomycin, ciprofloxacin, and linezolid was assessed using the Etest (bioMérieux). MICs for susceptibility (S), intermediate susceptibility (I), and resistance (R) were defined according to the recommendations of the European Committee on Antimicrobial Susceptibility Testing (EUCAST). For this study, frozen isolates were plated on Columbia agar plates with 5% sheep blood (bioMérieux) and harvested for further DNA extraction.

### Whole-genome sequencing.

Genomic DNA isolation, WGS, assembly, and contig filtering were performed as described previously (16). High-molecular-weight (HMW) DNA was isolated from blood cultures using the MagAttract HMW DNA kit (Qiagen, Hilden, Germany) according to the manufacturer’s protocol for Gram stain-positive bacteria. Ready-to-sequence libraries were obtained with a NexteraXT kit (Illumina, Inc., San Diego, CA, USA). Paired-end sequencing (2 by 300 bp) was performed on a MiSeq instrument (Illumina, Inc.). Raw reads were *de novo* assembled into a draft genome using SPAdes (version 3.11.1) (17). Contigs were filtered for a minimum coverage of 5 and a minimum length of 200 bp. Sequencing quality was checked with FastQC. Sequencing generated 409,744 to 1,655,480 reads and a mean coverage of 35- to 136-fold (see Table S1 in the supplemental material).

### Core genome MLST.

A C. diphtheriae core genome multilocus sequence typing (cgMLST) scheme was generated with the SeqSphere^+^ target definer tool (version 6.0.0; Ridom, Muenster, Germany) (18). We used strain NCTC 13129 as a seed genome (NCBI accession no. NC_002935.2) and 22 complete C. diphtheriae genomes as query sequences (NCBI accession no. NC_016782.1, NC_016783.1, NC_016785.1, NC_016786.1, NC_016787.1, NC_016788.1, NC_016789.1, NC_016790.1, NC_016799.1, NC_016800.1, NC_016801.1, NC_016802.1, NZ_CP018331, NZ_CP020410.2, NZ_CP025209.1, NZ_CP029644.1, NZ_LN831026.1, NZ_LR134537.1, NZ_LR134538.1, NZ_CP038504.1, CP038789.1, and LT990688.1) and applied default software parameters. A 1,439-locus cgMLST scheme and a 747-locus accessory target scheme were obtained.

### *tox* and *dtxR* allele library definition.

Two allele libraries were created with SeqSphere^+^ by downloading all available complete sequences from the NCBI: one for the DT gene (*tox*) and one for the DT regulator gene (*dtxR*). Putative *tox* and *dtxR* sequences from the isolates were extracted if they had at least 90% homology with one of the library sequences. Sequences were aligned using the ClustalW algorithm (R version 3.6.1, MSA package) (19, 20). DNA sequences were translated into amino acid sequences using the NCBI blastx tool with default parameters. Alignment figures were obtained using the R package MSA and the Latex package Texshade (21).

### Analysis.

Single nucleotide polymorphism (SNP) analysis of sequences was performed with CSI Phylogeny 1.4, available from the Center for Genomic Epidemiology (22), using strain NCTC 13129 as a reference genome. We used the online tool using ribosomal MLST (rMLST) (https://pubmlst.org/species-id) on assembled genomes of isolates identified as C. diphtheriae bv. *mitis*/bv. *belfanti* by biochemical investigation performed upon reception (see “Samples,” above). We used the results to segregate them between C. diphtheriae bv. *mitis*, *C. belfantii*, and C. rouxii. Isolates were characterized by MLST and cgMLST using SeqSphere^+^. A minimum spanning tree (MST) and a neighbor-joining tree (NJT) were computed using the number of cgMLST allelic differences among the isolates. Antibiotic resistance genes (ARGs) were identified using the ResFinder tool available from the Center for Genomic Epidemiology (23). Statistical analysis was performed using R (package epitools). Dendrograms were created from a distance matrix (from SNP analysis or cgMLST) with the R packages MSA and dendextend. Histograms and dot plots were created with R (package ggplot2).

### Data availability.

Raw reads were deposited in the Sequence Read Archive (SRA) under project accession number PRJNA608828. Biosamples were assigned BioSample accession no. SAMN14209767 to SAMN14209827, and raw-read sequences were assigned SRA accession no. SRR11184121 to SRR11184181. Assembly files were deposited on the GenBank database and assigned accession numbers JADQTP000000000.1 to JADQVX000000000.1.

## RESULTS

### Sample characteristics.

A total of 57 C. diphtheriae strains isolated between 2011 and 2019 from 55 different patients were included in this study (see Table S1 in the supplemental material). Three patients had mixed infections with Staphylococcus aureus, and one had a mixed infection with S. aureus, Streptococcus pyogenes, and Arcanobacterium haemolyticum. Two to 13 C. diphtheriae strains were isolated every year in Austria. The majority of the samples were sent by medical facilities located in the province of Vienna, 1 out of the 9 Austrian provinces with a population of 1.8 million people (Fig. S1A). Burgenland was the only province with no reported cases over this period. Isolates belonged to C. diphtheriae bv. *mitis*, C. diphtheriae bv. *gravis*, and *C. belfantii*, with various frequencies over the study period (Fig. S1B). No strain belonged to the recently described species *C. rouxii* (24). Out of 57 isolates, the DT gene was detected by PCR for only 3 of them (all C. diphtheriae bv. *mitis*). If a negative PCR result is enough to classify an isolate as nontoxigenic, an Elek test is needed to prove that a strain is toxigenic (25). Unfortunately, no Elek test was performed for any of the Austrian isolates. Therefore, the three isolates positive by PCR are referred to as “*tox*^+^” in this article.

For each isolate, the MLST profile was determined by *in silico* extraction from WGS data. Seven unknown alleles (4 *dnaK*, 1 *fusA*, 1 *leuA*, and 1 *odhA*) and 12 unknown sequence types (STs) were submitted to the *Corynebacterium* database of PubMLST (https://pubmlst.org/cdiphtheriae). Newly described STs were assigned ST652 to ST663. In total, 34 different STs were identified.

**FIG 1:**
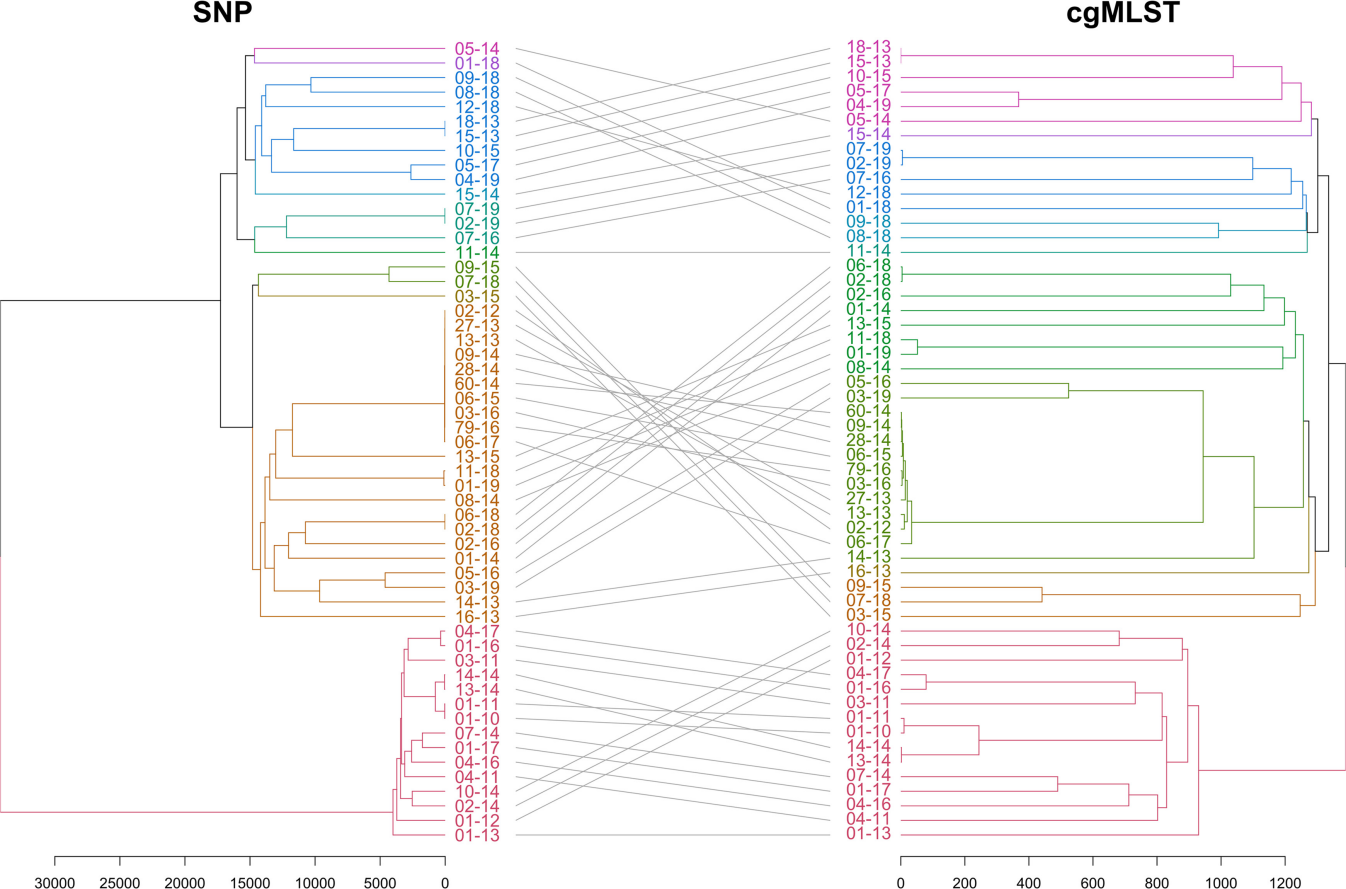
Dendrogram computed from the distance matrix from SNP analysis (left) and cgMLST analysis (right). Gray lines connect isolates in both trees. Scale bars indicate the numbers of differences between the isolates, in SNPs (left) or allelic differences (right).

To assess the diversity among the isolate sequences, we performed a genome-wide SNP analysis (Fig. 1, left). The maximum number of SNP pair counts between two isolates was 34,187, and sequences were segregated into seven main branches.

### cgMLST analysis.

**FIG 2:**
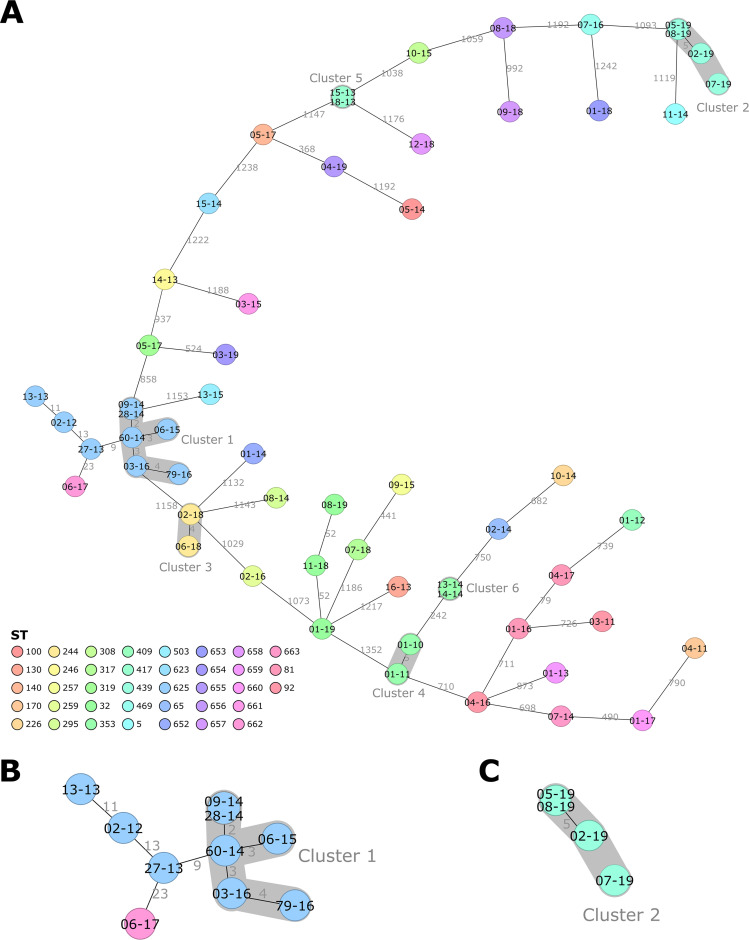
Minimum spanning tree (MST) computed using the number of allelic differences from local C. diphtheriae cgMLST. Isolates identifiers are indicated on the nodes. Distances between the isolates (number of allelic differences) are indicated on the connecting lines. Nodes are colored according to isolate ST. Clusters with fewer than 5 allelic differences are shaded in dark gray and named (cluster 1 and cluster 2, etc.). The MST includes all 57 isolates (A), 10 isolates from cluster 1 and the surrounding area (B), and 3 isolates from cluster 2 (C).

We designed an *ad hoc* cgMLST scheme and compared the distances between the isolates (in allelic differences) with the ones obtained by SNP analysis (Fig. 1). The main branches were conserved using both methods. Despite having a lower discriminatory power than SNP analysis, cgMLST analysis seems to be globally representative of the genetic diversity of our isolates. An MST was computed from cgMLST data (Fig. 2A). We found up to 1,389 allelic differences in the 1,439 targets of the core genome. Using a cluster threshold of five allelic differences (26), six clusters were identified.

Cluster 1 was the largest cluster identified, comprising six isolates obtained from five different patients (isolates 09-14 and 28-14 came from the same patient) (Fig. 2B). All patients were diagnosed in Vienna but over a period of 3 years (2014 to 2016). Even though they did not belong to cluster 1, isolates 27-13, 02-12, and 13-13 showed a low number of allelic differences. They were also isolated in Vienna but in 2012 to 2013. Cluster 2 included four different patients (Fig. 2C), all diagnosed in Vienna in 2019. For these two clusters, no epidemiological link between the cases was documented.

The four remaining clusters included only two isolates each. Clusters 3 and 4 included two samples from the same patient (but collected at different dates). Isolate 06-18 from cluster 3 was obtained 2 weeks after isolate 02-18, as a postantibiotic therapy control. The two isolates from cluster 4 were obtained 5 months apart. Differences between these isolates (4 and 5 allelic differences for clusters 3 and 4, respectively) underlined genetic divergence within the host. For cluster 5, an epidemiological link between the two patients was established (direct contact). The two patients of cluster 6 came from the same family.

### Link with clinical data.

To assess whether the genetic characteristics of the isolates correlated with clinical features, we compared our findings with patient data. For samples obtained after 2013, patients’ clinical and demographic data were available (Table S2). Patients were mainly men (41/48) and were distributed among all age groups (from 1 month to 88 years old). Clinical information allowed us to segregate patients into three disease types: skin infection (e.g., ulcer or infected wound), respiratory infection (e.g., rhinitis, angina, sinusitis, or suspected diphtheria), and other infection (one patient with spondylodiscitis). The majority of patients had skin infections (34/48), and 14/48 had respiratory infections (including 1 with no sex/age data) (Table S2). There seemed to be differences between age groups, with an increased frequency of skin disease in adults (20 to 60 years old).

**FIG 3:**
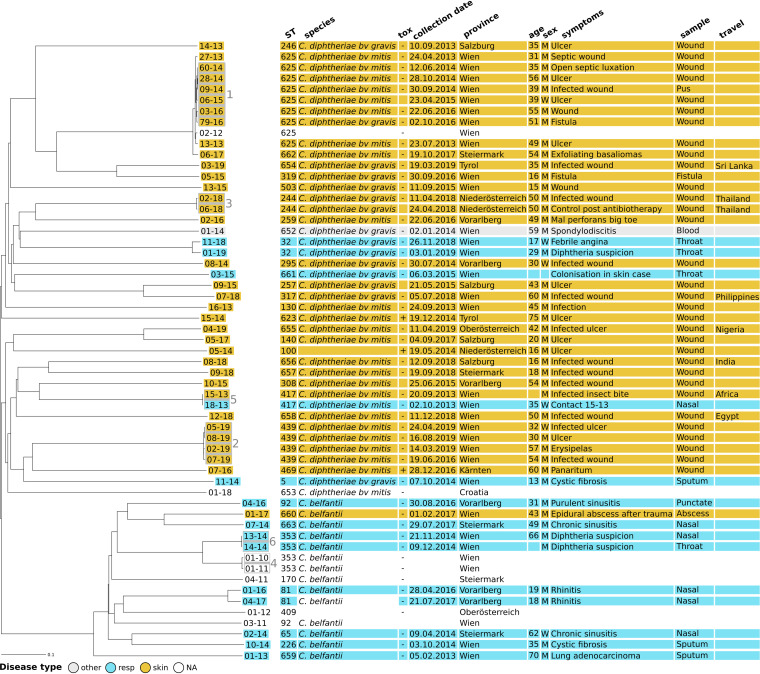
Neighbor-joining tree computed using the number of allelic differences from local C. diphtheriae cgMLST. The scale bar indicates the percentage of allelic differences between the isolates. Colors indicate patient disease types: respiratory infection, skin infection, and other infection. Columns on the right provide additional information on isolates (ST, species, *tox* status, date of collection of the sample [day.month.year], province of isolation, and type of sample used for isolation) and on the patients (age, sex, symptoms, and recent travel history). M, man; W, woman; NA, not applicable.

Disease type data were combined with data from cgMLST analysis in a neighbor-joining tree (NJT) (Fig. 3). The NJT revealed that one branch (bottom of the tree) had a higher proportion of respiratory disease. This branch corresponded to *C. belfantii* isolates. We tested this association in a univariate analysis model using respiratory disease as an outcome and type of organism (C. diphtheriae bv. *mitis*, C. diphtheriae bv. *gravis*, and *C. belfantii*) as exposure (Table 1). In spite of the small sample size described here, we found that patients with respiratory infection were 57 times more likely to be infected by *C. belfantii*, whereas C. diphtheriae bv. *mitis* was associated with skin infection.

**TABLE 1:** Numbers and frequencies of isolates exhibiting specific microbiological properties, depending on the patient disease type (respiratory or skin infection)^*a*^

Disease type	All		Respiratory infection		Skin infection		OR (95% CI)	*P* value
	No. of isolates	% of isolates	No. of isolates	% of isolates	No. of isolates	% of isolates		
*tox* status								
+	3	6	0		3	10		
−	48	94	14	100	28	90		

Species								
*C. belfantii*	13	25	9	64	1	3	57.60 (5.95–557.99)	3.73E−06
C. diphtheriae bv. *mitis*	25	48	1	7	23	70	0.03 (0.00–0.29)	1.10E−04
C. diphtheriae bv. *gravis*	14	27	4	29	9	27	1.07 (0.27–4.28)	0.94

All	55	100	14	25	34	62		

a Odds ratios (OR), 95% confidence intervals (CI), and *P* values (Wilcoxon test) were calculated by univariate analysis using the type of infection as an outcome (respiratory as a positive outcome and skin as a negative outcome) and species as exposure (target species as positive exposure and the other two species as negative exposure).

Eight patients had documented history of travel to a country where diphtheria is endemic (Fig. 3, right). Among them, four strains (08-18, 12-18, 03-19, and 04-19) corresponded to a newly described ST and were isolated recently (2018 to 2019). One patient with recent travel to Thailand was infected with ST244 (02-18/06-18), which was involved in a Thai outbreak in 2012 and could have kept circulating until 2018 (27). Strain 07-18 might have been imported from the Philippines, as a German patient infected with the same ST317 in 2010 had also traveled to the Philippines (PubMLST data). Isolate 15-13 belonged to ST417, which is more commonly found in Russia. The history of travel to Africa of this patient might be unrelated to his infection.

### Antimicrobial resistance.

**FIG 4:**
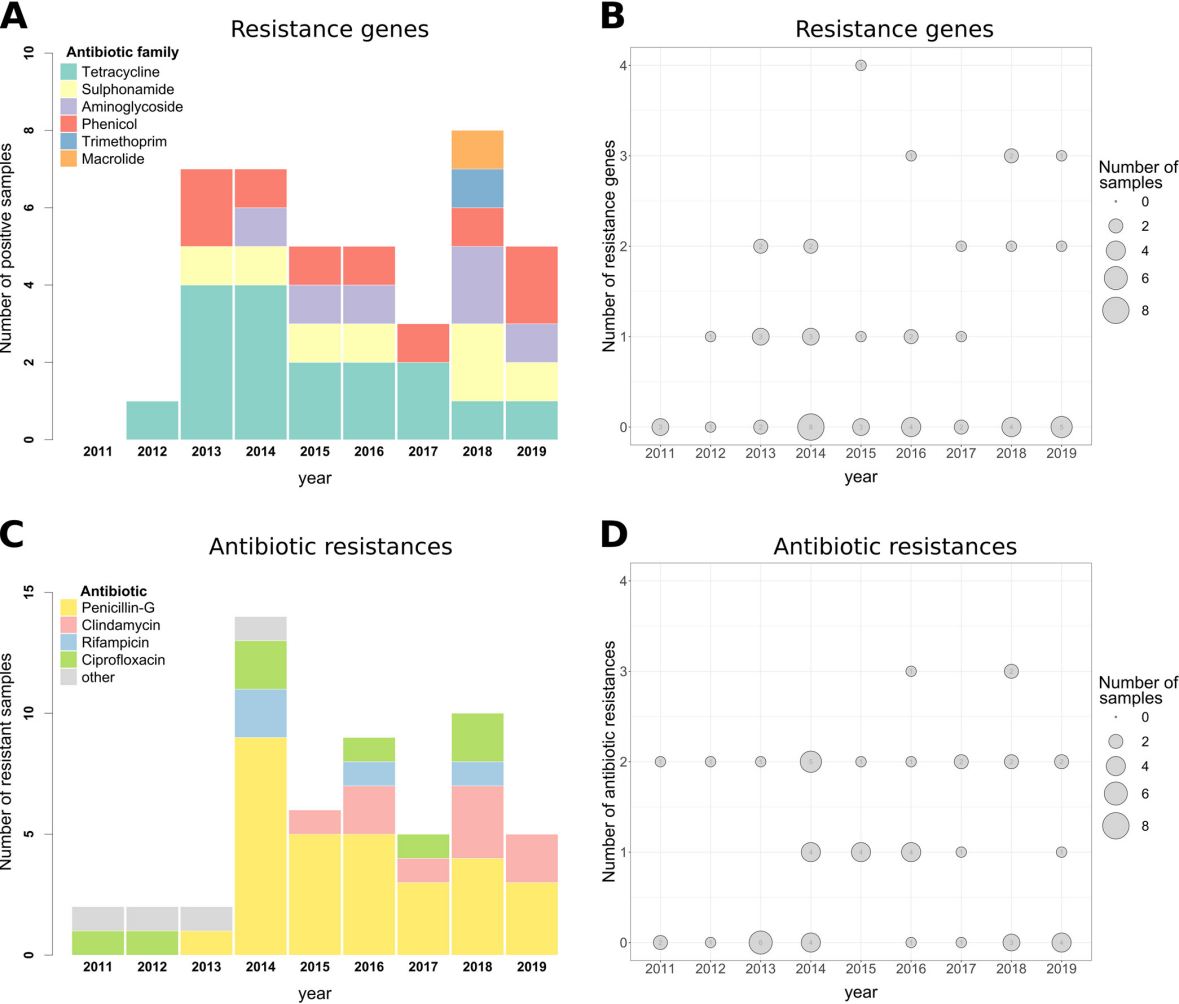
(A) Number of isolates per year containing antibiotic resistance genes against each antibiotic family. Colors correspond to the antibiotic families. (B) Number of antibiotics against which antibiotic resistance genes were identified in the isolate, depending on the year of isolation. The size of the dots (and number in each dot) indicates the number of isolates. (C) Number of isolates per year exhibiting antimicrobial resistance against different antibiotics. Colors correspond to the antibiotics tested. (D) Number of antibiotics against which antimicrobial resistance was identified, depending on the year of isolation. The size of the dots (and number in each dot) indicates the number of isolates.

Using the Center for Genomic Epidemiology tool ResFinder, we searched our sequence data for ARGs (Table S3). Many ARGs identified were targeting tetracycline, but ARGs against five other antibiotic families were also found. Figure 4A illustrates the number of strains carrying ARGs over time. Every year, around half of the isolates had at least one ARG (Fig. 4B). ARGs for at least two different antibiotic families were found in 30% of the isolates, and one isolate from 2015 carried ARGs against four different antibiotic families. Over 90% of strains isolated from patients with respiratory disease had no ARGs, whereas this was the case for only 31% of skin infection isolates (Table S4). This higher frequency of AMR might be due to the localization of infection (skin/respiratory tract) but also to *C. belfantii* and C. diphtheriae phylogenetic differences, as *C. belfantii* isolates were associated with respiratory infections.

AMR against antibiotics of clinical relevance was also tested using the Etest (bacterial culture in the presence of variable antibiotic concentrations). C. diphtheriae isolates showed AMR against penicillin, clindamycin, and ciprofloxacin mostly (Fig. 4C). More than half of the isolates were resistant to penicillin, which is one of the standard treatments for *Corynebacterium* infection (Table S5). Frequent penicillin resistance has already been observed in other studies (27–30). Two or three types of AMR were found in 35% of the isolates (Fig. 4D). In most cases, penicillin AMR was combined with clindamycin or ciprofloxacin AMR. Unlike what was observed for the ARGs, AMR was identified in strains isolated from both respiratory and skin infections (Table S4).

### Diphtheria toxin gene.

Toxigenic properties of C. diphtheriae are carried by the DT gene *tox*. The transcription of *tox* is controlled by the *dtxR* regulon (31). Only three isolates were *tox*^+^ by PCR, but nontoxigenic *tox*-bearing (NTTB) strains have been reported (32). Therefore, we decided to search for *tox* and *dtxR* in our isolates’ sequences.

**FIG 5:**
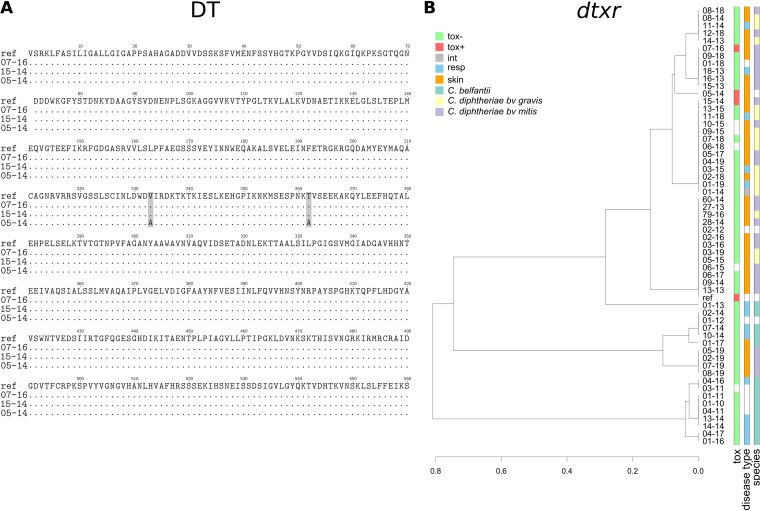
(A) Alignment of amino acid sequences corresponding to putative *tox* genes extracted from isolate sequences (3 sequences). Divergent amino acids are highlighted in gray. (B) Phylogenetic analysis of putative *dtxR* genes extracted from isolate sequences (57 sequences). Bars on the right indicate the *tox* status, disease type, and bacterial species of each isolate. Isolates were compared to reference strain NCTC 13129.

We found only 3 putative *tox* sequences, in the three *tox*^+^ isolates (05-14, 15-14, and 07-16). We aligned them with the toxigenic reference strain NCTC 13129 and found variants at five positions (Fig. S2). Nucleotide sequences were translated to amino acid sequences and aligned. Only two nucleotide variants led to changes in the respective amino acids (Fig. 5A). Translated *tox* sequences of isolates 15-14 and 07-16 correspond to the reference DT sequence under UniProt accession no. P00588.2, and the translated *tox* sequence of isolate 05-14 corresponds to the reference DT sequence under NCBI accession no. WP_181997018.1.

For the *dtxR* gene, putative sequences were identified in all 57 isolates (Fig. 5B). The diversity within the *dtxR* sequences was low, which is coherent with its global regulatory function (33). Alignment of these sequences showed no specific segregation depending on *tox* or disease type. However, the *C. belfantii* isolates’ *dtxR* sequences seemed to be grouped together, as previously shown for the seven housekeeping genes of the MLST scheme or by genomic analysis (14, 34).

## DISCUSSION

Austrian C. diphtheriae strains isolated over the past 10 years showed high genetic variability. This diversity is not so surprising given the long period of time during which isolates were distributed and the likelihood of imported cases. However, previous studies in other countries pointed out the predominance of a type (*C. belfantii* in France [35] and C. diphtheriae bv. *mitis* in Algeria [29]) or even a single ST (ST76 in Vancouver, Canada [36]). This was not the case in our study. Twelve isolates had previously undescribed STs, among which four corresponded to patients with a documented history of travel to a country of endemicity. This suggested that regions of endemicity might play an important role in bacterial genetic divergence and the emergence of new subtypes.

Except for two Viennese clusters (clusters 1 and 2), no clear distribution of the isolates according to time or place was found. Cluster 1 included five patients diagnosed between 2014 and 2016, but no further strains belonging to this ST have been identified since then. Isolates 27-13, 02-12, and 13-13, although not in cluster 1, were very similar. They might be related to cluster 1, with divergence over time explaining the higher number of allelic differences. Cluster 2 included four isolates from 2019, which were the only ones from ST439. These two clusters suggested either local circulation of C. diphtheriae or a common importation route. None of the available epidemiological data had linked these cases together. Therefore, WGS analysis could be a tool to complement epidemiological investigations. Routine WGS of C. diphtheriae isolates would allow investigators to identify clusters in real time, launch further investigations, increase awareness at a local level, and implement control measures.

The system for the classification of C. diphtheriae isolates into different biovars is still broadly used but has been largely criticized (34, 37–39). Genomic analyses have failed to show any correlation of biovars with STs or with genes involved in the corresponding metabolic pathways (34, 39, 40). In our study, we did not identify specific patterns of distribution of C. diphtheriae isolates depending on their classification as C. diphtheriae bv. *mitis* or bv. *gravis*, adding to previous evidence questioning the relevance of this classification. On the other hand, specific clustering of C. diphtheriae bv. *belfanti* led to a change of its taxonomic status to *C. belfantii* (14). In our study, a strong correlation between *C. belfantii* and respiratory diseases was found, as previously described. Whether this is due to phylogenic specificities or metabolic properties is still undetermined.

We looked for antimicrobial resistance by combining a genetic approach (searching WGS data for known ARGs) and classical phenotypic resistance testing. The microbiological approach is more robust but also time- and resource-consuming, so it is performed only for antibiotics of clinical relevance. On the other hand, the WGS approach allows a wider search for ARGs. However, not all AMR mechanisms are identified, so some types of AMR can be undetected. In addition, the identification of an ARG does not necessarily prove that the strain is resistant. Therefore, the two approaches are complementary, and combining them brings out a more precise picture of the AMR profile. The first discrepancy between WGS and Etest data came from penicillin AMR: although 55% of the isolates were penicillin resistant, no β-lactam ARGs were found in their sequences. Such differences were previously noticed in other studies (36). It also underlines the issue of defining breakpoints for AMR. Indeed, breakpoints used in diagnostic laboratories are usually the ones defined by international organizations (EUCAST or CLSI) and might not fit the distribution of MICs among the population or the clinical relevance of AMR. Previous studies have suggested that a breakpoint of 0.125 μg/ml for penicillin in C. diphtheriae, as used in our routine laboratory, might be too low (27, 36, 41). Such an overclassification of isolates as penicillin resistant can lead to changes in antibiotic prescriptions toward other antibiotics, such as erythromycin, which are usually less well tolerated (36). Using the WGS approach, we found ARGs against tetracycline and sulfonamides in 31% and 13% of strains, respectively, especially skin isolates. This observation is consistent with those of previous studies; however, no explanation regarding why such types of AMR were found has been given so far, as these antibiotics are not used to treat *Corynebacterium* infections (27, 36). In Austria, these two antibiotic families are mostly used in animal medicine (42). If other *Corynebacterium* species are considered zoonotic, very few cases of C. diphtheriae carriage or infection in animals have been described (43, 44). The interface between animal and human *Corynebacterium* strains could be an explanation for the high prevalence of tetracycline and sulfonamide ARGs in skin isolates, but this would require further studies.

Most of the isolates included in this study were *tox* negative by PCR, with only 3 *tox*^+^ isolates. However, PCR indicates the presence of a *tox* gene but not whether it encodes a functional DT. We were able to extract putative *tox* genes from whole-genome sequences of the three *tox*^+^ isolates only. All of them matched published DT sequences, indicating that the three *tox*^+^ isolates can encode a functional DT. If DT expression cannot be demonstrated using such genetic analyses, this sequence analysis of *tox* can identify and discard some false-positive results (*tox*^+^ by PCR but negative by an Elek test). For the *dtxR* gene, we found homologous sequences in all of our samples, as previously described (45). These *dtxR* sequences were not very divergent, which might indicate that this gene is under stronger selective pressure than the rest of the genome. *C. belfantii dtxR* genes were grouped together, which is in agreement with previous studies showing that *dtxR* sequences vary among *Corynebacterium* species but not within them (45).

To conclude, this retrospective analysis of C. diphtheriae in Austria using WGS showed high genetic variability among the strains. We confirmed the strong association of *C. belfantii* with respiratory infections. Our data also underlined the complementarity of genetic and microbiological methods for AMR identification. Extracting *tox* genes from WGS data supported PCR results on the toxigenicity of the isolates. Overall, this study shows how beneficial implementing WGS in C. diphtheriae surveillance would be. It would allow the identification of clusters that have not been highlighted by epidemiological investigations, complement AMR data with additional antibiotic families, and remove some false-negative results of toxigenicity obtained by PCR. Sequences could also feed larger studies on C. diphtheriae, improving our global understanding of this bacterium.

## Supplementary Material

Supplemental file 1
