# Supplementary material for: Assessing the Genetic Diversity of Austrian Corynebacterium diphtheriae Clinical Isolates, 2011 to 2019
Source: J Clin Microbiol. 2021 Feb 18;59(3):e02529-20. doi: 10.1128/JCM.02529-20 (PMC8106727; doi:10.1128/JCM.02529-20)
Supplement: Supplemental file 1 [file JCM.02529-20-s0001.pdf]

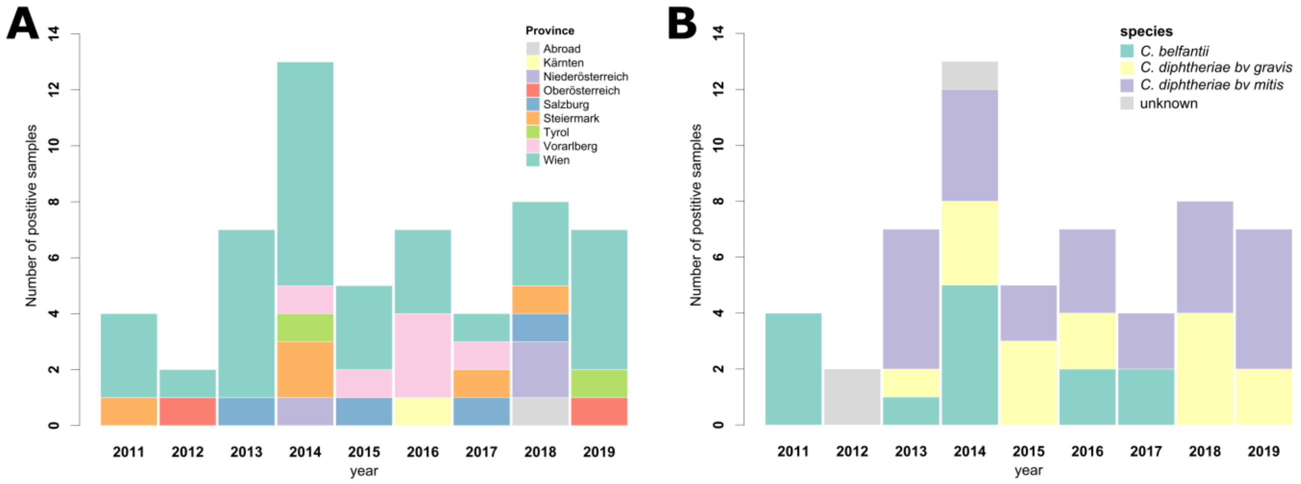

Figure S1. Number of isolates per year, depending on the province of isolation (A) or the isolate species (B).

```
              10        20        30        40        50        60        70        80        90
ref   GTGAGCAGAAAACTGTTTGCGTCAATCTTAATAGGGGCGCTACTGGGGATAGGGGCCCCACCTTCAGCCCATGCAGGCGCTGATGATGTT
07-16 ..........................................................................................
15-14 ..........................................................................................
05-14 ..........................................................................................
             100       110       120       130       140       150       160       170       180
ref   GTTGATTCTTCTAAATCTTTTGTGATGGAAAACTTTTCTTCGTACCACGGGACTAAACCTGGTTATGTAGATTCCATTCAAAAAGGTATA
07-16 ..........................................................................................
15-14 ..........................................................................................
05-14 ..........................................................................................
             190       200       210       220       230       240       250       260       270
ref   CAAAAGCCAAAATCTGGTACACAAGGAAATTATGACGATGATTGGAAAGGGTTTTATAGTACCGACAATAAATACGACGCTGCGGGATAC
07-16 ..........................................................................................
15-14 ..........................................................................................
05-14 ..........................................................................................
             280       290       300       310       320       330       340       350       360
ref   TCTGTAGATAATGAAAACCCGCTCTCTGGAAAAGCTGGAGGCGTGGTCAAAGTGACGTATCCAGGACTGACGAAGGTTCTCGCACTAAAA
07-16 ..........................................................................................
15-14 ..........................................................................................
05-14 ..........................................................................................
             370       380       390       400       410       420       430       440       450
ref   GTGGATAATGCCGAAACTATTAAGAAAGAGTTAGGTTTAAGTCTCACTGAACCGTTGATGGAGCAAGTCGGAACGGAAGAGTTTATCAAA
07-16 ..........................................................................................
15-14 ......................................................C...................................
05-14 ......................................................C...................................
             460       470       480       490       500       510       520       530       540
ref   AGGTTCGGTGATGGTGCTTCGCGTGTAGTGCTCAGCCTTCCCTTCGCTGAGGGGAGTTCTAGCGTTGAATATATTAATAACTGGGAACAG
07-16 ..........................................................................................
15-14 ..........................................................................................
05-14 ..........................................................................................
             550       560       570       580       590       600       610       620       630
ref   GCGAAAGCGTTAAGCGTAGAACTTGAGATTAATTTTGAAACCCGTGGAAAACGTGGCCAAGATGCGATGTATGAGTATATGGCTCAAGCC
07-16 ..........................................................................................
15-14 ..........................................................................................
05-14 ..........................................................................................
             640       650       660       670       680       690       700       710       720
ref   TGTGCAGGAAATCGTGTCAGGCGATCAGTAGGTAGCTCATTGTCATGCATAAATCTTGATTGGGATGTCATAAGGGATAAAACTAAGACA
07-16 ..........................................................................................
15-14 ..........................................................................A...............
05-14 ...................................................................C......................
             730       740       750       760       770       780       790       800       810
ref   AAGATAGAGTCTTTGAAAGAGCATGGCCCTATCAAAAATAAAATGAGCGAAAGTCCCAATAAAACAGTATCTGAGGAAAAAGCTAAACAA
07-16 ..........................................................................................
15-14 ..........................................................................................
05-14 ...............................................................G..........................
             820       830       840       850       860       870       880       890       900
ref   TACCTAGAAGAATTTCATCAAACGGCATTAGAGCATCCTGAATTGTCAGAACTTAAAACCGTTACTGGGACCAATCCTGTATTCGCTGGG
07-16 ..........................................................................................
15-14 ..........................................................................................
05-14 ..........................................................................................
             910       920       930       940       950       960       970       980       990
ref   GCTAACTATGCGGCGTGGGCAGTAAACGTTGCGCAAGTTATCGATAGCGAAACAGCTGATAATTTGGAAAAGACAACTGCTGCTCTTTCG
07-16 ..........................................................................................
15-14 ..........................................................................................
05-14 ..........................................................................................
            1000      1010      1020      1030      1040      1050      1060      1070      1080
ref   ATACTTCCTGGTATCGGTAGCGTAATGGGCATTGCAGACGGTGCCGTTCACCACAATACAGAAGAGATAGTGGCACAATCAATAGCTTTA
07-16 ..........................................................................................
15-14 ..........................................................................................
05-14 ..........................................................................................
            1090      1100      1110      1120      1130      1140      1150      1160      1170
ref   TCGTCTTTAATGGTTGCTCAAGCTATTCCATTGGTAGGAGAGCTAGTTGATATTGGTTTCGCTGCATATAATTTTGTAGAGAGTATTATC
07-16 ..........................................................................................
15-14 ..........................................................................................
05-14 ..........................................................................................
            1180      1190      1200      1210      1220      1230      1240      1250      1260
ref   AATTTATTTCAAGTAGTTCATAATTCGTATAATCGTCCCGCGTATTCTCCGGGGCATAAAACACAACCATTTCTTCATGACGGGTATGCT
07-16 ..............................................................G...........................
15-14 ..............................................................G...........................
05-14 ..............................................................G...........................
            1270      1280      1290      1300      1310      1320      1330      1340      1350
ref   GTCAGTTGGAACACTGTTGAAGATTCGATAATCCGAACTGGTTTTCAAGGGGAGAGTGGGCACGACATAAAAATTACTGCTGAAAATACC
07-16 ..........................................................................................
15-14 ..........................................................................................
05-14 ..........................................................................................
            1360      1370      1380      1390      1400      1410      1420      1430      1440
ref   CCGCTTCCAATCGCGGGTGTCCTACTACCGACTATTCCTGGAAAGCTGGACGTTAATAAGTCCAAGACTCATATTTCCGTAAATGGTCGG
07-16 ..........................................................................................
15-14 ..........................................................................................
05-14 ..........................................................................................
            1450      1460      1470      1480      1490      1500      1510      1520      1530
ref   AAAATAAGGATGCGTTGCAGAGCTATAGACGGTGATGTAACTTTTTGTCGCCCTAAATCTCCTGTTTATGTTGGTAATGGTGTGCATGCG
07-16 ..........................................................................................
15-14 ..........................................................................................
05-14 ..........................................................................................
            1540      1550      1560      1570      1580      1590      1600      1610      1620
ref   AATCTTCACGTGGCATTTCACAGAAGCAGCTCGGAGAAAATTCATTCTAATGAAATTTCGTCGGATTCCATAGGCGTTCTTGGGTACCAG
07-16 ..........................................................................................
15-14 ..........................................................................................
05-14 ..........................................................................................
            1630      1640      1650      1660      1670      1680
ref   AAAACAGTAGATCACACCAAGGTTAATTCTAAGCTATCGCTATTTTTTGAAATCAAAAGCTGA
07-16 ...............................................................
15-14 ...............................................................
05-14 ...............................................................
```

Figure S2. Alignment of putative *tox* genes extracted from isolate sequences (3 sequences). Divergent nucleotides are highlighted in grey. Isolates were compared with reference strain NCTC 13129.

sample ID	collection date	reception date	province of isolation	sample type	species	tox	ST	read count	coverage	N50	cgMLST good targets (%)
**01-10**		10.01.2011	Wien		*C. belfantii*	-	353	918906	79	31366	95.9
**01-11**		12.05.2011	Wien		*C. belfantii*	-	353	720546	62	28492	95.3
**03-11**		12.08.2011	Wien		*C. belfantii*	*NA*	92	1184492	93	28851	96.2
**04-11**		09.09.2011	Steiermark		*C. belfantii*	-	170	1052502	81	26979	96.4
**01-12**		04.12.2012	Oberösterreich		*C. diphtheriae/belfantii*	-	409	828224	80	33929	97.0
**02-12**		19.12.2012	Wien		*C. diphtheriae/belfantii*	-	625	437280	35	129105	99.3
**01-13**	05.02.2013	05.02.2013	Wien	Sputum	*C. belfantii*	-	659	876756	70	32758	96.2
**27-13**	24.04.2013	24.04.2013	Wien	Wound	*C. diphtheriae bv. mitis*	-	625	1091502	103	111273	99.4
**13-13**	23.07.2013	23.07.2013	Wien	Wound	*C. diphtheriae bv. mitis*	-	625	1346368	120	129257	99.4
**14-13**	10.09.2013	11.09.2013	Salzburg	Wound	*C. diphtheriae bv. gravis*	-	246	1280080	118	131688	99.1
**15-13**	20.09.2013	23.09.2013	Wien	Wound	*C. diphtheriae bv. mitis*	-	417	952264	83	100609	99.1
**16-13**	24.09.2013	23.09.2013	Wien	Wound	*C. diphtheriae bv. mitis*	-	130	1263480	114	99686	99.1
**18-13**	02.10.2013	03.10.2013	Wien	Nasal	*C. diphtheriae bv. mitis*	-	417	1109430	94	121563	99.1
**01-14**	02.01.2014	03.01.2014	Wien	Blood	*C. diphtheriae bv. gravis*	-	652	858988	69	153731	99.2
**02-14**	09.04.2014	09.04.2014	Steiermark	Nasal	*C. belfantii*	-	65	1135826	114	33033	96.9
**05-14**	19.05.2014	27.05.2014	Niederösterreich	Wound	*C. diphtheriae/belfantii*	+	100	1079114	100	178240	99.3
**60-14**	12.06.2014	17.06.2014	Wien	Wound	*C. diphtheriae bv. mitis*	-	625	1351542	123	129260	99.3
**07-14**	29.07.2017	30.07.2014	Steiermark	Nasal	*C. belfantii*	-	663	1179390	94	38592	96.8
**08-14**	30.07.2014	31.07.2014	Vorarlberg	Wound	*C. diphtheriae bv. gravis*	-	295	1147230	99	254950	98.8
**09-14**	30.09.2014	01.10.2014	Wien	Pus	*C. diphtheriae bv. mitis*	-	625	1106782	104	129260	99.3
**10-14**	03.10.2014	04.10.2014	Wien	Sputum	*C. belfantii*	-	226	1116368	83	35554	96.5
**11-14**	07.10.2014	08.10.2014	Wien	Sputum	*C. diphtheriae bv. gravis*	-	5	1095430	106	100474	98.4
**28-14**	28.10.2014	31.10.2014	Wien	Wound	*C. diphtheriae bv. mitis*	-	625	1041064	99	129260	99.1
**13-14**	21.11.2014	25.11.2014	Wien	Nasal	*C. belfantii*	-	353	1012444	82	32919	96.1
**14-14**	09.12.2014	10.12.2014	Wien	Throat	*C. belfantii*	-	353	1195372	94	32834	96.2
**15-14**	19.12.2014		Tyrol	Wound	*C. diphtheriae bv. mitis*	+	623	1076412	95	137940	99.0
**03-15**	06.03.2015	17.03.2015	Wien	Throat	*C. diphtheriae bv. gravis*	-	661	908350	87	133280	98.6
**06-15**	23.04.2015	24.04.2015	Wien	Wound	*C. diphtheriae bv. mitis*	*NA*	625	764712	78	108128	99.3
**09-15**	21.05.2015	22.05.2015	Salzburg	Wound	*C. diphtheriae bv. gravis*	*NA*	257	919486	98	177066	99.4
**10-15**	25.06.2015	30.06.2015	Vorarlberg	Wound	*C. diphtheriae bv. mitis*	*NA*	308	931800	92	224351	99.2
**13-15**	11.09.2015	14.09.2015	Wien	Wound	*C. diphtheriae bv. gravis*	-	503	598924	58	112858	98.9
**01-16**	28.04.2016	02.05.2016	Vorarlberg	Nasal	*C. belfantii*	-	81	1043450	94	48155	96.7
**02-16**	22.06.2016	23.06.2016	Vorarlberg	Wound	*C. diphtheriae bv. mitis*	-	259	1133066	102	137239	98.9
**03-16**	22.06.2016	23.06.2016	Wien	Wound	*C. diphtheriae bv. mitis*	-	625	1104886	46	108142	99.1
**04-16**	30.08.2016	31.08.2016	Vorarlberg	Punctate	*C. belfantii*	-	92	729238	62	33662	96.7
**05-16**	30.09.2016	03.10.2016	Wien	Fistula	*C. diphtheriae bv. gravis*	-	319	961494	94	156805	99.3
**79-16**	02.10.2016	07.10.2016	Wien	Wound	*C. diphtheriae bv. gravis*	-	625	879708	87	137135	99.2
**07-16**	28.12.2016	30.12.2016	Kärnten	Wound	*C. diphtheriae bv. mitis*	+	469	788280	77	148055	99.0
**01-17**	01.02.2017	02.02.2017	Wien	Abscess	*C. belfantii*	-	660	409744	36	34213	96.9
**04-17**	21.07.2017	23.07.2017	Vorarlberg	Nasal	*C. belfantii*	-	81	918272	75	36741	96.7
**05-17**	04.09.2017	05.09.2017	Salzburg	Wound	*C. diphtheriae bv. mitis*	-	140	1056928	91	112001	98.3
**06-17**	19.10.2017	23.10.2017	Steiermark	Wound	*C. diphtheriae bv. mitis*	-	662	1240336	114	98636	99.3
**01-18**		19.03.2018	Croatia		*C. diphtheriae bv. mitis*	-	653	736490	69	117897	99.0
**02-18**	11.04.2018	12.04.2018	Niederösterreich	Wound	*C. diphtheriae bv. gravis*	-	244	890980	78	152127	99.2
**06-18**	24.04.2018	25.04.2018	Niederösterreich	Wound	*C. diphtheriae bv. gravis*	*NA*	244	899916	91	147328	99.2
**07-18**	05.07.2018	05.07.2018	Wien	Wound	*C. diphtheriae bv. gravis*	-	317	872990	92	189040	99.2
**08-18**	12.09.2018	12.09.2018	Salzburg	Wound	*C. diphtheriae bv. mitis*	-	656	907582	68	100184	99.3
**09-18**	19.09.2018	20.09.2018	Steiermark	Wound	*C. diphtheriae bv. mitis*	-	657	956234	80	118599	98.7
**11-18**	26.11.2018	27.11.2018	Wien	Throat	*C. diphtheriae bv. gravis*	-	32	1036876	95	160492	99.0
**12-18**	11.12.2018	11.12.2018	Wien	Wound	*C. diphtheriae bv. mitis*	-	658	1286296	110	140717	98.6
**01-19**	03.01.2019	03.01.2019	Wien	Throat	*C. diphtheriae bv. gravis*	-	32	889250	75	53703	98.3
**02-19**	14.03.2019	18.03.2019	Wien	Wound	*C. diphtheriae bv. mitis*	-	439	1092504	95	140931	99.0
**03-19**	19.03.2019	20.03.2019	Tyrol	Wound	*C. diphtheriae bv. gravis*	-	654	828406	62	50928	99.3
**04-19**	11.04.2019	11.04.2019	Oberösterreich	Wound	*C. diphtheriae bv. mitis*	-	655	750200	62	91896	98.5
**05-19**	24.04.2019	25.04.2019	Wien	Wound	*C. diphtheriae bv. mitis*	-	439	808374	40	140351	98.7
**07-19**	19.06.2016	19.06.2019	Wien	Wound	*C. diphtheriae bv. mitis*	-	439	1519656	124	140754	99.0
**08-19**	16.08.2019	20.08.2019	Wien	Wound	*C. diphtheriae bv. mitis*	-	439	1196788	56	141061	98.7

Table S1. Descriptive characteristics of study isolates (N=57), in term of sample data (date of isolation, province of isolation, sample type), microbiological characteristics (species, tox, ST) and sequencing quality (number of reads, estimated coverage, N50, good targets in cgMLST). Blanks and NAs correspond to missing data.

Disease type		all	resp		skin		OR [95% CI]	pvalue
		#	#	%	#	%		
Sex	man	42	10	24%	31	74%	REF	0.20
	woman	6	3	50%	3	50%	**3.10** [0.54-17.87]	
Age (years)	<20	11	5	45%	6	55%	REF	0.32
	20-40	14	4	29%	10	71%	**0.48** [0.09-2.52]	
	40-60	17	1	6%	15	88%	**0.08** [0.01-0.84]	
	>60	6	3	50%	3	50%	**1.20** [0.16-8.80]	
**All**		**55**	**14**	**25%**	**34**	**62%**		

Table S2. Patient demographics (number # and frequency %) depending on the patient disease type (respiratory or skin infection). Odds ratio (OR), 95% confidence interval (95% CI) and p values (wilcoxon test) were calculated using univariate analysis.

Sample ID	Tetracyclin	Sulphonamide	Aminoglycoside	Phenicol	Trimethoprim	Macrolide
**01-10**	-	-	-	-	-	-
**03-11**	-	-	-	-	-	-
**04-11**	-	-	-	-	-	-
**01-12**	-	-	-	-	-	-
**02-12**	tet(O)	-	-	-	-	-
**01-13**	-	-	-	-	-	-
**27-13**	tet(O)	-	-	-	-	-
**13-13**	tet(O)	-	-	-	-	-
**14-13**	-	sul1	-	-	-	-
**15-13**	tet(33)	-	-	cmx	-	-
**16-13**	-	-	-	-	-	-
**18-13**	tet(33)	-	-	cmx	-	-
**01-14**	-	-	-	-	-	-
**02-14**	-	-	-	-	-	-
**05-14**	-	-	-	-	-	-
**60-14**	tet(O)	-	-	-	-	-
**07-14**	-	-	-	-	-	-
**08-14**	-	-	aph(3')-Ia	cmx	-	-
**09-14**	tet(O)	-	-	-	-	-
**10-14**	-	-	-	-	-	-
**11-14**	-	-	-	-	-	-
**28-14**	tet(O)	-	-	-	-	-
**13-14**	-	-	-	-	-	-
**14-14**	-	-	-	-	-	-
**15-14**	tet(W)	sul1	-	-	-	-
**03-15**	-	-	-	-	-	-
**06-15**	tet(O)	-	-	-	-	-
**09-15**	-	-	-	-	-	-
**10-15**	tet(O)	sul1	aph(3')-Ia/aph(6)-Id	cmx	-	-
**13-15**	-	-	-	-	-	-
**01-16**	-	-	-	-	-	-
**02-16**	-	-	-	-	-	-
**03-16**	tet(O)	-	-	-	-	-
**04-16**	-	-	-	-	-	-
**05-16**	-	-	-	-	-	-
**79-16**	tet(O)	-	-	-	-	-
**07-16**	-	sul1	aph(3')-Ia/aph(6)-Id	cmx	-	-
**01-17**	-	-	-	-	-	-
**04-17**	-	-	-	-	-	-
**05-17**	tet(O)	-	-	cmx	-	-
**06-17**	tet(O)	-	-	-	-	-
**01-18**	-	-	-	-	-	-
**02-18**	tet(33)	sul1	-	-	-	-
**07-18**	-	-	-	-	-	-
**08-18**	-	-	aph(3')-Ia/aph(6)-Id	-	dfrA1	erm(X)
**09-18**	-	sul1	aph(3')-Ia/aph(6)-Id	cmx	-	-
**11-18**	-	-	-	-	-	-
**12-18**	-	-	-	-	-	-
**01-19**	-	-	-	-	-	-
**02-19**	-	-	-	-	-	-
**03-19**	-	sul1	aph(3')-Ia/aph(6)-Id	cmx	-	-
**04-19**	tet(O)	-	-	cmx	-	-
**05-19**	-	-	-	-	-	-
**07-19**	-	-	-	-	-	-
**08-19**	-	-	-	-	-	-

Table S3. Antibiotic resistance genes found in the isolate sequences, classified by antibiotic family.

Disease type		all		resp		skin	
		**#**	**%**	**#**	**%**	**#**	**%**
Resistance genes	Tetracycline	16	29%	1	7%	15	44%
	Sulphonamide	7	13%	0	-	7	21%
	Aminoglycoside	6	11%	0	-	6	18%
	Phenicol	9	16%	1	7%	8	24%
	Trimethoprim	1	2%	0	-	1	3%
	Macrolide	1	2%	0	-	1	3%
Resistance	Penicillin-G	30	55%	10	71%	20	59%
	Clindamycin	9	16%	2	14%	7	21%
	Rifampicin	4	7%	1	7%	3	9%
	Ciprofloxacin	8	15%	4	29%	2	6%
	other	4	7%	1	7%	1	3%
**All**		**55**	**100%**	**14**	**25%**	**34**	**62%**

Table S4. Number (#) and frequency (%) of isolates containing antibiotic resistance genes or exhibiting antimicrobial resistances, depending on the patient disease type (respiratory or skin infection).

Sample ID	Penicillin-G *R > 0,125 µg/mL*	Clindamycin *R > 0,55 µg/mL*	Rifampicin *R > 0,5 µg/mL* *I > 0,06 µg/mL*	Ciprofloxacin *R > 1 µg/mL*	other
**01-10**	-	-	-	-	-
**03-11**	-	-	-	R	Moxifloxacin
**04-11**	-	-	-	-	-
**01-12**	-	-	-	-	-
**02-12**	-	-	-	R	Amikacin
**01-13**	-	-	-	-	-
**27-13**	-	-	-	-	-
**13-13**	-	-	-	-	-
**14-13**	-	-	-	-	-
**15-13**	-	-	-	-	-
**16-13**	-	-	-	-	-
**18-13**	R	-	-	-	Aminopenicillin
**01-14**	-	-	-	-	-
**02-14**	R	-	-	-	-
**05-14**	R	-	-	-	Aminopenicillin
**60-14**	R	-	-	-	-
**07-14**	R	-	I	-	-
**08-14**	R	-	R	-	-
**09-14**	-	-	-	-	-
**10-14**	R	-	-	-	-
**11-14**	R	-	-	-	-
**28-14**	-	-	-	-	-
**13-14**	R	-	-	R	-
**14-14**	R	-	-	R	-
**15-14**	-	-	-	-	-
**03-15**	R	R	-	-	-
**06-15**	R	-	-	-	-
**09-15**	R	-	-	-	-
**10-15**	R	-	-	-	-
**13-15**	R	-	-	-	-
**01-16**	-	-	-	R	-
**02-16**	R	-	-	-	-
**03-16**	-	-	-	-	-
**04-16**	R	R	-	-	-
**05-16**	R	R	I	-	-
**79-16**	R	-	-	-	-
**07-16**	R	-	-	-	-
**01-17**	R	-	-	-	-
**04-17**	R	-	-	R	-
**05-17**	R	R	-	-	-
**06-17**	-	-	-	-	-
**01-18**	-	-	-	-	-
**02-18**	R	R	I	-	-
**07-18**	R	R	-	-	-
**08-18**	R	R	-	R	-
**09-18**	R	-	-	R	-
**11-18**	-	-	-	-	-
**12-18**	-	-	-	-	-
**01-19**	-	-	-	-	-
**02-19**	R	-	-	-	-
**03-19**	R	R	-	-	-
**04-19**	R	R	-	-	-
**05-19**	-	-	-	-	-
**07-19**	-	-	-	-	-
**08-19**	-	-	-	-	-
**% resistant isolates**	**55%**	**16%**	**7%**	**15%**	

Table S5. Antimicrobial resistances of the isolates. R resistant; I intermediate; - susceptible. Antibiotic tested for all isolates were Penicillin-G, Clindamycin, Rifampicin, Ciprofloxacin, Vancomycin and Linezolid. Isolates from 2010 to 2013 were also tested for Aminopenicillin, Aminopenicillin + β-lactamase inhibitor, Imipenem, Cefazolin, Gentamicin, Amikacin, Erythromycin and Moxifloxacin.
